# Predicting 30-Day Outcomes in Primary Intracerebral Hemorrhage Using the Intracerebral Hemorrhage Score: A Study in Bangladesh

**DOI:** 10.7759/cureus.73227

**Published:** 2024-11-07

**Authors:** Swapan Kumar Ray, Mohammad Sadekur Rahman Sarkar, K M Ahasan Ahmed, Mashfiqul Hasan, Tareq Esteak, Mohammad Nur Uddin, Junaid Abdullah Jamiul Alam, F M Monjur Hasan, Md. Tauhidul Islam Chowdhury, Md. Badrul Alam Mondal

**Affiliations:** 1 Neurology, National Institute of Neurosciences and Hospital, Dhaka, BGD; 2 Endocrinology and Metabolism, Bangabandhu Sheikh Mujib Medical University, Dhaka, BGD; 3 Neurology, Ad-din Sakina Women's Medical College, Jashore, BGD

**Keywords:** 30-day mortality, glasgow coma scale, ich score, intracerebral hemorrhage, short-term outcome

## Abstract

Introduction

Intracerebral hemorrhage (ICH) is a severe subtype of stroke associated with high rates of mortality and morbidity. Accurate early prognostication is essential for optimizing treatment strategies and improving patient outcomes. The ICH score, which includes clinical and imaging variables, is widely used to predict mortality and functional outcomes in ICH patients. However, limited data on the applicability of this scoring system are available from low-income countries. This study aims to evaluate the ICH score as a predictor of 30-day mortality and functional outcome, as measured by the modified Rankin Scale (mRS), in ICH patients at a tertiary care hospital in Bangladesh.

Methods

We conducted a prospective cohort study over one year at the National Institute of Neurosciences and Hospital in Dhaka, Bangladesh. One hundred patients aged over 18 years with confirmed primary ICH were enrolled. We collected data on demographics, clinical presentation, risk factors, and imaging findings, including the Glasgow Coma Scale (GCS), ICH score components, and hematoma volume. Patients were followed up for 30 days, and outcomes were assessed using the mRS. Statistical analyses included univariate and multivariate logistic regression and Kaplan-Meier survival estimates.

Results

A total of 100 participants were enrolled for the study. The mean age of participants was 59.2 ± 14.5 years, and 57 were men, and 43 were women. The overall 30-day mortality rate was 44%. Mortality rates increased significantly with higher ICH scores (p < 0.001), with all patients scoring 4 or 5 on the ICH score dying within 30 days. Lower GCS scores, larger hematoma volumes, the presence of intraventricular hemorrhage (IVH), and increasing age were associated with higher mortality. Multivariate analysis identified increasing age (p = 0.023), lower GCS score (p = 0.003), and higher ICH score (p = 0.025) as independent predictors of 30-day mortality. Higher ICH scores were also significantly associated with poor functional outcomes (mRS ≥ 4) at both discharge and 30 days (p < 0.001). GCS score emerged as an independent predictor of poor functional outcomes at 30 days (p = 0.012).

Conclusions

The ICH score is an effective tool for predicting 30-day mortality and functional outcomes in patients with primary ICH. Incorporating the ICH score and GCS assessment into routine clinical practice can aid healthcare providers in early risk stratification, optimizing treatment plans, and improving resource allocation.

## Introduction

Intracerebral hemorrhage (ICH), a subtype of stroke, frequently causes significant mortality and morbidity among adults [1]. ICH has an annual incidence of 10-30 per 100,000 population, contributing to two million (10%-15%) of the approximately 15 million strokes worldwide each year [2]. According to a survey of stroke patients, the prevalence of stroke in Bangladesh is 1.14% [3]. Asian populations exhibit a higher incidence of ICH and associated mortality and morbidity than Western populations [4]. Spontaneous, nontraumatic ICH refers to intraparenchymal bleeding that may extend into the ventricles and, rarely, into the subarachnoid space [5]. We classify spontaneous ICH as primary or secondary based on the underlying cause of bleeding. Uncontrolled hypertension (HTN) and cerebral amyloid angiopathy primarily cause primary ICH, which is responsible for 78%-88% of cases. Secondary ICH results from vascular malformations (arteriovenous malformations and aneurysms), tumors, and the use of anticoagulant medications [5].

ICH has a very high mortality rate, and survivors often have significant long-term disabilities. The 30-day mortality of ICH patients is nearly 40%, making it one of the deadliest acute medical events [6]. Therefore, accurate outcome prognostication is critical in managing patients with primary ICH [7]. Early and precise prognostic assessment facilitates the optimization of treatment plans and minimizes the risk of overtreatment [8]. It is important to provide patients and their families with a personalized assessment of survival likelihood after ICH [9]. Prognostic models, such as the ICH score, confer greater accuracy than relying solely on clinical judgment. Prognostic scores based on large data sets allow physicians to make more objective predictions.

Over time, researchers have developed and updated many scoring systems to predict outcomes in patients with spontaneous intracerebral hemorrhage. The ICH score, a prognostic tool with a maximum of 6 points, considers five independent variables: the Glasgow Coma Scale (GCS), ICH volume, the presence of intraventricular hemorrhage (IVH), ICH origin, and patient age (≥80 years) [10]. Clinicians can obtain all variables during the initial evaluation through GCS assessment and imaging investigations such as computed tomography (CT) scans. Researchers developed the score as a predictive tool for 30-day mortality.

Subsequent studies have evaluated the ICH score as a predictor of long-term functional outcomes [10]. Other studies have assessed its role as a predictor of in-hospital mortality and discharge outcomes in spontaneous ICH [11]. Although different studies report varying mortality rates, most have correlated the ICH score with in-hospital mortality, morbidity at discharge, and 30-day and 12-month functional outcomes [4].

A prognostic model such as the ICH score is particularly important in low- to middle-income countries such as Bangladesh, where healthcare access, imaging technologies, and early intervention facilities are limited. A CT scan is the mainstay of investigation to diagnose ICH worldwide. Even though a CT scan is not cheap, it is quite available in our country. ICH score can be calculated only with a CT scan without further investigations. Though the ICH score has been validated in the Western population, very few studies in Bangladesh have examined its role as an outcome predictor. So, the validation of the ICH score in the Bangladeshi population is necessary due to differences in healthcare infrastructure, treatment protocols, and population characteristics. Due to the poor long-term outcomes of spontaneous ICH patients, accurate prognostication is crucial for optimizing treatment plans and resource allocation, especially in low- and middle-income countries.

Therefore, this study aimed to assess the ICH score as a predictor of 30-day mortality and functional outcome, as measured by the modified Rankin Scale (mRS), in patients with intracerebral hemorrhage at a tertiary care hospital in Bangladesh.

## Materials and methods

We conducted this prospective cohort study in the Stroke Unit of the National Institute of Neurosciences and Hospital, Dhaka, over one year from July 2021 to June 2022. All patients above 18 years of age admitted to the Stroke Unit within 24 hours of symptom onset with non-contrast CT evidence of spontaneous ICH were enrolled in the study. Suspected cases of secondary ICH (intracranial space-occupying lesions with bleeding, arteriovenous malformations, aneurysms, or hemorrhagic transformation of ischemic stroke) were excluded by neuroimaging (MRI/CT angiography/CT venography of the brain) and other laboratory investigations. Patients on anticoagulation therapy and post-traumatic ICH were also excluded from the study.

We obtained formal ethical approval from the Ethical Review Committee of the National Institute of Neurosciences and Hospital before starting the study (approval number: IRB/NINS/2020/80.1). After selecting the participants according to the inclusion and exclusion criteria, we obtained informed written consent from each participant or their legal guardian. We conducted history taking, focusing on clinical variables, risk factors, and physical examination as per standard protocol. We managed hemorrhagic stroke per prevailing stroke guidelines promptly. All patients underwent routine hematological tests (complete blood count, random blood glucose, serum creatinine, and serum electrolytes), electrocardiogram, chest X-ray, urine routine examination, and head CT scan. We recorded demographic data, including age, sex, prior medical history, relevant comorbidities (HTN and diabetes), risk factors such as smoking and alcohol consumption, and the use of antiplatelet or anticoagulant medications.

We collected data relevant to the ICH score, encompassing GCS, patient age, hemorrhage location, the presence of IVH, and bleeding volume. The GCS score at the time of admission was used. We used initial brain CT scan findings to assess hemorrhagic characteristics. We calculated hemorrhage volume using the ABC/2 formula, where A is the longest diameter, B is the diameter perpendicular to A, and C is the number of slices multiplied by their thickness. We categorized the hematoma location based on supratentorial and infratentorial compartments. Supratentorial locations included lobar, thalamic, internal capsule, and basal ganglia regions. Infratentorial involvement comprised cerebellar and brainstem hemorrhages. IVH refers to bleeding within the ventricles of the brain. The presence or absence of IVH was also recorded on the initial brain CT scan. All parameters of the brain CT scan were recorded by a physician. We monitored patients via follow-up for up to one month.

The modified Rankin Scale (mRS) was used to assess the functional status of the patients. mRS is a commonly used stroke outcome scale with scores ranging from 0 (no symptoms at all) to 6 (dead). mRS score was obtained at discharge and at 30 days post-ictus, either during direct follow-up visits in the outpatient department or over the telephone by a researcher. An mRS score of ≥4 was considered a poor outcome.

Statistical analysis

We conducted data analysis using IBM SPSS Statistics for Windows, version 23.0 (IBM Corp., Armonk, NY). We performed exploratory data analysis to describe the study population, summarizing continuous variables using central tendency and dispersion measures such as mean and standard deviation (SD). We expressed qualitative or categorical variables as frequencies and proportions. We used the chi-square test to determine the association between categorical variables. We analyzed predictors of short-term outcomes using logistic regression analysis. We assessed Kaplan-Meier estimates between different groups using the log-rank test and generated cumulative mortality curves for survival analysis. We considered a p-value of less than 0.05 to be statistically significant.

## Results

One hundred patients diagnosed with primary ICH were enrolled in the study. The mean age of the participants was 59.2 (±14.5 years standard deviation {SD}), and 57 were men (Table 1). A history of HTN was present in 89 participants; 22 had diabetes, and nine had a history of taking antiplatelet medications. Eighty-two patients presented with acute hemiparesis, 75 with altered consciousness, and 65 with vomiting. The mean systolic blood pressure (BP) was 156.5 ± 28.4 mmHg, and the mean diastolic BP was 92.9 ± 15.2 mmHg. The median GCS score was 9 (range: 7-11). Most participants (71 of 100) had a GCS score between 5 and 12, 19 had a score between 3 and 4, and 10 had a score between 13 and 15. The mean ICH score was 2.06 ± 1.17 (SD). Thirty-four participants had an ICH score of 2, followed by 31 with a score of 1, 15 with a score of 3, 13 with a score of 4, five with a score of 0, and two with a score of 5. None of the participants had an ICH score of 6. The mean duration of hospital stay was 6.0 ± 2.8 days (calculated for those who survived during the hospital course; n = 70).

**Table 1:** Demographic and clinical parameters of the study participants (n = 100) ICH, intracerebral hemorrhage; GCS, Glasgow Coma Scale; HTN, hypertension

Characteristics		Frequency, N (%)
Sex	Male	57 (57.0%)
	Female	43 (43.0%)
Clinical findings	Acute hemiparesis	82 (82.0%)
	Altered level of consciousness	75 (75.0%)
	Vomiting	65 (65.0%)
	Speech difficulty	21 (21.0%)
	Severe headache	9 (9.0%)
	Seizure	8 (8.0%)
	History of HTN	89 (89.0%)
	History of diabetes	22 (22.0%)
	History of smoking	14 (14.0%)
	History of taking antiplatelet	9 (9.0%)
GCS category	13-15	10 (10.0%)
	5-12	71 (71.0%)
	3-4	19 (19.0%)
ICH score category	0	5 (5.0%)
	1	31 (31.0%)
	2	34 (34.0%)
	3	15 (15.0%)
	4	13 (13.0%)
	5	2 (2.0%)

The most common site of hemorrhage was the basal ganglia (65 of 100), followed by the thalamus (n = 15), lobar regions (n = 11), cerebellum (n = 6), and pons (n = 3) (Table 2). The mean hematoma volume was 21.4 ± 19.3 mL. Ninety-one participants had a supratentorial hemorrhage, 72 had a blood volume of less than 30 mL, and 54 had a ventricular extension.

**Table 2:** Imaging findings of the study participants (n = 100)

Characteristics		Frequency, N (%)
Site of hemorrhage	Basal ganglia	65 (65.0%)
	Thalamus	15 (15.0%)
	Lobar	11 (11.0%)
	Cerebellum	6 (6.0%)
	Pons	3 (3.0%)
Volume	≥30 mL	28 (28.0%)
	<30 mL	72 (72.0%)
Origin	Supratentorial	91 (91.0%)
	Infratentorial	9 (9.0%)
Intraventricular extension		54 (54.0%)

**Figure 1:**
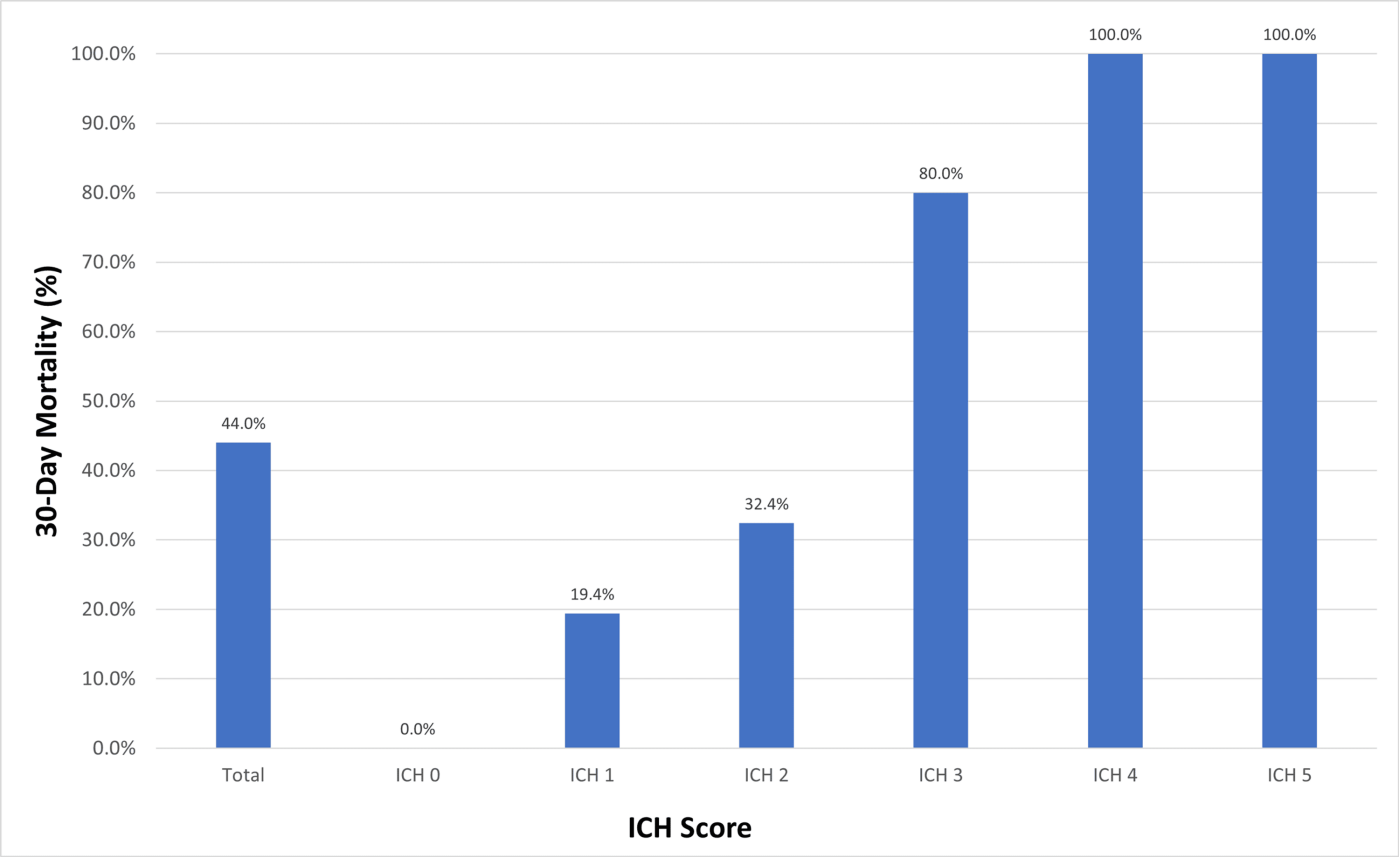
Overall 30-day mortality associated with individual ICH score ICH: intracerebral hemorrhage

The overall 30-day mortality rate was 44.0%. Mortality rates for ICH scores of 1, 2, and 3 were 19.4%, 32.4%, and 80.0%, respectively. None of the participants with an ICH score of 0 died, while all participants with scores of 4 or 5 died. Mortality increased with higher ICH scores, which was statistically significant (p < 0.001 via chi-square test; Figure 1).

**Figure 2:**
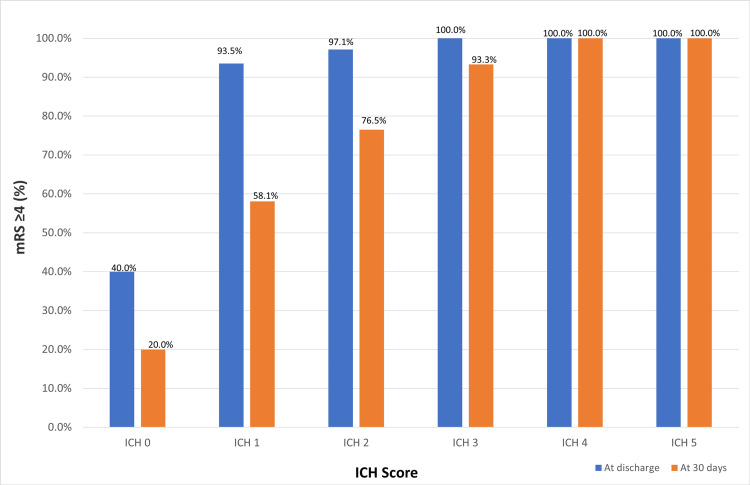
The ICH score and percentage of patients with mRS score of ≥4 at discharge and at 30 days ICH, intracerebral hemorrhage; mRS, modified Rankin Scale

A significant relationship was found between ICH score and poor functional outcomes, defined as an mRS score of ≥ 4, at discharge and at 30 days. At discharge, poor functional outcomes occurred in 40.0% of the participants with an ICH score of 0, 93.5% with a score of 1, 97.1% with a score of 2, and 100% with scores of 3, 4, and 5. At 30 days, poor functional outcomes were observed in 20.0% of the participants with an ICH score of 0, 58.1% with a score of 1, 76.5% with a score of 2, 93.3% with a score of 3, and 100% with scores of 4 and 5. The increase in poor functional outcomes with higher ICH scores was statistically significant at both discharge (p < 0.001) and 30 days (p = 0.001, via chi-square test; Figure 2).

Univariate logistic regression revealed that increasing age (p = 0.004), systolic BP at admission (p = 0.024), diastolic BP at admission (p = 0.009), lower GCS score (p < 0.001), larger hematoma volume (p < 0.001), the presence of IVH (p = 0.013), and higher ICH score (p < 0.001) were significantly associated with 30-day mortality. Multivariate analysis showed that increasing age (p = 0.023), lower GCS score (p = 0.003), and higher ICH score (p = 0.025) were independent predictors of 30-day mortality (Table 3).

**Table 3:** Logistic regression analysis for predictors of 30‑day mortality SBP, systolic blood pressure; CI, confidence interval; DBP, diastolic blood pressure; GCS, Glasgow Coma Scale; ICH, intracerebral hemorrhage; IVH, intraventricular hemorrhage; NA, not applicable

Parameters	Univariate		Multivariate	
	P-value	Odds ratio (95% CI)	P-value	Odds ratio (95% CI)
Age, per year increase	0.004	1.047 (1.015-1.080)	0.023	1.056 (1.008-1.108)
Male sex	0.435	1.376 (0.617-3.071)	NA	NA
Hypertension	0.918	0.936 (0.266-3.295)	NA	NA
Diabetes	0.876	1.078 (0.417-2.793)	NA	NA
Antiplatelet medication	0.165	2.789 (0.656-11.858)	NA	NA
SBP, per mmHg increase	0.024	1.018 (1.002-1.033)	0.928	0.998 (0.965-1.033)
DBP, per mmHg increase	0.009	1.039 (1.010-1.070)	0.538	1.018 (0.962-1.078)
GCS	<0.001	0.513 (0.399-0.659)	0.003	0.619 (0.454-0.845)
Infratentorial origin	0.468	1.667 (0.420-6.617)	NA	NA
ICH volume, per mL increase	<0.001	1.080 (1.039-1.122)	0.982	1.001 (0.935-1.071)
IVH	0.013	2.857 (1.251-6.527)	0.217	0.301 (0.045-2.028)
ICH score, per unit increase	<0.001	4.938 (2.609-9.347)	0.025	5.339 (1.240-22.99)

Univariate logistic regression for predicting poor functional outcomes at 30 days showed that increasing age (p = 0.019), lower GCS score (p < 0.001), larger hematoma volume (p = 0.004), and higher ICH score (p < 0.001) were associated with poor functional outcomes. Multivariate regression analysis revealed that only the GCS score (p = 0.012) was an independent predictor of poor functional outcomes at 30 days (Table 4).

**Table 4:** Logistic regression analysis for predictors of poor functional outcome (mRS ≥ 4) at 30 days mRS, modified Rankin Scale; SBP, systolic blood pressure; CI, confidence interval; DBP, diastolic blood pressure; GCS, Glasgow Coma Scale; ICH, intracerebral hemorrhage; IVH, intraventricular hemorrhage; NA, not applicable

Parameter	Univariate		Multivariate	
	P-value	Odds ratio (95% CI)	P-value	Odds ratio (95% CI)
Age, per year increase	0.019	1.043 (1.007-1.079)	0.088	1.039 (0.994-1.085)
Male sex	0.587	0.777 (0.311-1.936)	NA	NA
Hypertension	0.919	1.076 (0.263-4.404)	NA	NA
Diabetes	0.483	0.690 (0.245-1.944)	NA	NA
SBP, per mmHg increase	0.325	1.008 (0.992-1.025)	NA	NA
DBP, per mmHg increase	0.326	1.015 (0.985-1.046)	NA	NA
GCS	<0.001	0.629 (0.508-0.778)	0.012	0.717 (0.554-0.929)
Infratentorial origin	0.307	0.330 (0.039-2.775)	NA	NA
ICH volume, per mL increase	0.004	1.081 (1.025-1.141)	0.429	1.027 (0.961-1.098)
IVH	0.068	2.347 (0.939-5.867)	NA	NA
ICH score, per unit increase	<0.001	3.393 (1.791-6.428)	0.220	1.700 (0.729-3.966)

**Figure 3:**
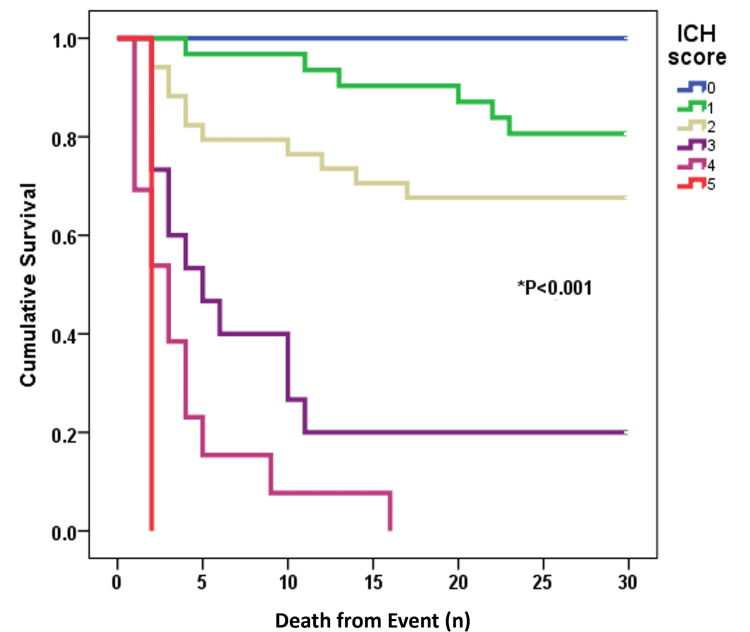
Kaplan-Meier survival curve from day 0 to day 30 showing cumulative survival of ICH patients with different ICH scores *P < 0.001 via log-rank test ICH: intracerebral hemorrhage

Kaplan-Meier survival curves demonstrated significant differences in survival times based on ICH scores. The median survival times for patients with ICH scores of 1, 2, 3, 4, and 5 were 13, four, three, three, and two days, respectively. None of the patients with an ICH score of 0 died. The survival curves clearly distinguished between the ICH scores, with statistically significantly lower survival times corresponding to higher ICH scores (p < 0.001 via log-rank test; Figure 3).

## Discussion

Stroke is a leading cause of death and acquired disability in adults worldwide. ICH accounts for 10%-15% of all strokes and presents a higher risk of morbidity and mortality compared to cerebral infarction and subarachnoid hemorrhage [12]. Over time, several scoring systems have been developed and refined to predict outcomes in patients with spontaneous ICH. The ICH score is a widely used system in high-income countries [13]. This study evaluated the utility of the ICH score in predicting 30-day mortality and functional outcomes in patients with primary ICH.

The 30-day mortality rates in our study for ICH scores of 0, 1, 2, and 3 were 0%, 19.4%, 32.4%, and 80.0%, respectively, while the rates for ICH scores of 4 and 5 were 100%. These results are consistent with prior research. Hemphill et al. reported mortality rates of 0%, 13%, 26%, 72%, 94%, and 100% for ICH scores of 0, 1, 2, 3, 4, and 5, respectively [13]. Ojha et al. found mortality rates of 16.7% for an ICH score of 0, 0% for a score of 1, 30% for a score of 2, 57.2% for a score of 3, 62.5% for a score of 4, and 100% for scores of 5 and 6 [4]. In line with previous studies, we found that higher ICH scores were associated with increased mortality. In both univariate (p < 0.001) and multivariate (p = 0.025) analyses, the ICH score was a statistically significant predictor of 30-day mortality.

The ICH score was also a reliable predictor of short-term (30-day) functional outcomes in patients with primary ICH [14]. In our study, higher ICH scores were associated with worse functional outcomes, defined as an mRS score of ≥4, both at discharge (p < 0.001) and at 30 days (p = 0.001). Univariate analysis identified age, GCS, hematoma volume, and ICH score as predictors of functional outcome, with GCS being the only independent predictor in multivariate analysis.

The GCS has been consistently identified as a strong predictor of mortality and morbidity in most previous studies [4,8,15]. In our study, lower GCS scores at admission were significantly associated with poor outcomes. GCS was a statistically significant predictor of 30-day mortality in both univariate (p < 0.001) and multivariate (p = 0.003) analyses. Additionally, GCS predicted functional outcomes in both univariate (p < 0.001) and multivariate (p = 0.012) analyses.

In our study, the majority of the participants (91 of 100) were under the age of 80, with a mean age of 59.2 ± 14.5 years. Men constituted 57.0% of the cohort. Rashid et al. reported that the peak incidence of ICH occurred in the 50-59 age group, with 79.1% of the patients being men [16]. Other studies also support these findings; Sarder et al. [17] reported a mean age of 58.7 ± 10.9 years, while Bhatia et al. [11] found a mean age of 57.3 ± 12.8 years, with 65.4% of the patients being men. In our study, increasing age was significantly associated with higher mortality (p = 0.023), a finding supported by studies by Kumari et al. [18] and Hegde et al. [15]. However, Ojha et al. found no such association [4].

HTN was the most common risk factor in our study, present in 89 of 100 participants, followed by diabetes (22 of 100), smoking (14 of 100), and antiplatelet use (nine of 100). These results are consistent with those of Rashid et al. [16]. Sarder et al. [17] reported HTN in 60.4% of the patients and diabetes in 56.3%, while Bhatia et al. [11] found HTN in 82.1% of the patients and diabetes in 15.8%. HTN remains the most significant risk factor for spontaneous ICH.

Regarding clinical presentation, most patients in our study had acute hemiparesis (82 of 100), altered consciousness (75 of 100), and vomiting (65 of 100). Additional symptoms included speech difficulty (21 of 100), severe headache (19 of 100), and seizures (eight of 100). Sarder et al. similarly reported hemiparesis in 60.4% of the patients, unconsciousness in 18.8%, and cerebellar symptoms in 12.5% [17]. Modi et al. also found that acute hemiparesis (69.7%) and altered sensorium (59.7%) were the most common presenting symptoms [19]. An altered sensorium is likely due to elevated intracranial pressure or the involvement of the reticular activating system in the brainstem.

In our univariate analysis, both systolic BP (p = 0.024) and diastolic BP (p = 0.009) were significantly associated with mortality, though this association did not persist in multivariate analysis. Hegde et al. [15] reported similar findings, whereas Ojha et al. [4] found no association between diastolic BP and mortality. Kumari et al. observed a significant association between diastolic BP and mortality but found no significant relationship with systolic BP [18].

In the current study, 65 of 100 participants had hematomas in the basal ganglia, 15 in the thalamus, 11 in the lobar regions, six in the cerebellum, and three in the pons. Bhatia et al. similarly observed hematomas in the basal ganglia (70.6%), thalamus (16.8%), lobar regions (4.2%), brainstem (7.0%), and cerebellum (1.4%) [11]. Sarder et al. also found that the basal ganglia was the most common site, followed by lobar regions, with other sites including the brainstem, thalamus, and cerebellum [17]. Other studies have reported comparable findings regarding hematoma locations [10,16].

The mean hospital stay in our study was 6.8 ± 2.3 days, which is consistent with the findings of Sarder et al. [17] but shorter than those reported by Rathor et al. [20]. The shorter hospital stays in our study may be attributed to hospital burden and the socioeconomic background of the patients.

Imaging findings play a critical role in predicting outcomes in patients with ICH. In our study, the mean hematoma volume was 21.4 ± 19.3 mL, and hematoma volume was significantly associated with 30-day mortality (p < 0.001) and functional outcome (p = 0.004). Additionally, the presence of IVH was significantly associated with mortality (p = 0.013), which is consistent with previous studies [4,13]. However, infratentorial bleeding was not associated with mortality (p = 0.468). Some studies have reported a significant association between infratentorial bleeding and mortality, while others have not [11]. Our study's limited number of patients with infratentorial hemorrhage (nine of 100) may explain the lack of association.

The 30-day mortality rate for spontaneous ICH ranges from 25% to 52%, with approximately half of these deaths occurring within 48 hours [11]. In our study, the 30-day mortality rate was 44.0%, with 30.0% of deaths occurring in the hospital. Islam et al. [21] reported a 30-day mortality rate of 38.0%, while Sarder et al. [17] found a rate of 27.1%. As our study was conducted at a neurology referral center, more acutely ill and severe cases of ICH were likely admitted, contributing to the higher mortality rate.

Adopting the ICH score and GCS for the prognostication of ICH patients can help improve patient care in resource-limited settings. By rapidly assessing patient severity, these scores can help prioritize care and optimize treatment plans. By quickly detecting high-risk patients, these tools can help allocate limited resources such as intensive care and specialized interventions to those most likely to benefit.

This study had several important limitations. First, the sample size was relatively small and drawn from a single tertiary care hospital, which may limit the generalizability of the findings to other settings or populations. Second, the study was observational and did not control for all potential confounding variables, such as variations in treatment protocols or rehabilitation services, which could have influenced patient outcomes. Third, the follow-up period was limited to 30 days, preventing the assessment of long-term mortality and functional outcomes beyond this timeframe. Additionally, some follow-up data were collected via telephone interviews, which may introduce recall bias or inaccuracies in reporting. Lastly, the study did not evaluate the impact of specific interventions or treatments received, making it difficult to assess how these factors may have affected the prognostic value of the ICH score and GCS. Furthermore, the variability of post-discharge care, such as rehabilitation and social support, might influence functional outcomes.

## Conclusions

On admission, the ICH score can effectively predict both 30-day mortality and functional outcomes in patients with primary ICH. Additionally, the GCS serves as an independent predictor of short-term outcomes, including mortality and morbidity, and increasing age is significantly associated with higher mortality in primary ICH. These findings suggest that integrating the ICH score and GCS assessments into routine clinical practice can aid healthcare providers in early risk stratification, optimizing treatment plans, and improving resource allocation. By utilizing these objective scoring systems, clinicians can offer personalized prognoses, enhance communication with patients and their families, and make informed decisions that may improve overall patient management and outcomes in primary ICH.
